# Evaluation of reference genes for quantitative PCR analysis during somatic embryogenesis in conifers

**DOI:** 10.1186/1753-6561-5-S7-O44

**Published:** 2011-09-13

**Authors:** José Javier de Vega-Bartol, Raissa Raquel Santos, Marta Simões, Celia Miguel

**Affiliations:** 1 Instituto de Biologia Experimental e Tecnológica/Instituto de Tecnologia Química e Biológica-Universidade Nova de Lisboa (IBET/ITQB-UNL), Portugal

## 

Clonal propagation by somatic embryogenesis (SE) has a great application potential in conifers, but its efficiency still needs further improvement. Quantitative PCR (qPCR) is widely used for gene expression analysis during SE as part of studies to characterize gene function. In qPCR, data is normalized against a reference gene with a uniform expression during the tested conditions. Studies have shown that no single housekeeping gene is universal for all experiments, so a systematic characterization of several endogenous genes for concrete conditions is fundamental for biological accuracy.

We evaluated 10 genes present in *Pinus pinaster* selected among those commonly used as endogenous reference in conifers (*actin*, *α-tubulin*, *elongation factor 1 α*, *adenosine kinase* and *ubiquitin*), previously tested in model plants (*Histone 3*, *SAND* protein and *clathrim adaptor complex* -CAC- subunit), and with a lower variation of expression in five RNA samples at different embryogenesis stages according to microarray hybridization results (homologous to a heat shock protein -HEATSK- and to an ether reductase -REDUC-) (Simões *et al*., unpublished results). Stability of the expression in three developmental stages (embryogenic tissue in proliferation, mature embryos and germinated embryos) was evaluated with two different algorithms (implemented by programs geNorm and Normfinder), both in *Pinus pinaster* and *Picea abies*, so results could be expanded to other conifers. Previously, efficiency was calculated with programs Linreg and qPCR miner and the distribution of Cts by statistical boxplot analysis, for each amplicon.

Results evidenced that the geometric averaging of two-to-four of these genes is accurate enough. Using genes *elongation factor 1 α*, *SAND* and *α-tubulin* as reference is the better generic combination, and so probably the preferable option to consider in other conifers. Among them, *SAND* and *α-tubulin* had a reduced range of Cts, while it was higher in *elongation factor 1 α*, especially in *Pinus pinaster*. Some discrepancies were detected in the ranking base on stability for the other genes, so this generic combination can be improved in certain stages or species, like *adenosine kinase* in *Pinus pinaster* or CAC when analysing embryogenic tissue in proliferation. *Adenosine kinase* and *ubiquitin* are also acceptable options, as they showed small ranges of Cts and medium stability values in the three stages in both species. By contrast, several genes showed a higher variation in our experiments, as both selected from the microarray analysis (REDUC and HEATSK), and one commonly used reference gene (*actin*), which evidence the importance of these kind of studies.

